# Ki 67 is a major, but not the sole determinant of Oncotype Dx recurrence score

**DOI:** 10.1038/bjc.2011.402

**Published:** 2011-10-04

**Authors:** S Sahebjam, R Aloyz, D Pilavdzic, M-L Brisson, C Ferrario, N Bouganim, V Cohen, W H Miller, L C Panasci

**Affiliations:** 1 Department of Medical Oncology, Jewish General Hospital, McGill University, 3755 Côte Ste-Catherine Road, Montreal, QC H3T 1E2, Canada; 2 Department of Pathology, Jewish General Hospital, McGill University, Montreal, QC H3T 1E2, Canada; 3 Segal Cancer Center, Lady Davis Institute, Jewish General Hospital, Montreal, QC H3T 1E2, Canada

**Keywords:** Ki 67 expression, immunohistochemistry, Oncotype Dx

## Abstract

**Background:**

Immunohistological assessment of Ki 67 expression is less expensive than Oncotype Dx, which is currently used to identify patients with lymph node-negative breast cancer, who will benefit from adjuvant chemotherapy.

**Methods:**

The relationship of immunohistologically measured Ki 67 to Oncotype DX recurrence score (RS) was examined in 53 cases of T1–2 N0 M0 (oestrogen receptor-positive, HER2/neu negative) breast cancer.

**Results:**

There was a strong linear correlation between Ki 67 value and the Oncotype Dx RS. All patients in the low Ki 67 group (Ki 67 of ⩽10%) had Oncotype Dx RSs of low or intermediate risk. The vast majority of patients (93.8%) in the high-Ki 67 group (Ki 67⩾25%) had oncotype RSs of high or intermediate risk.

**Conclusion:**

Ki 67 proliferation value is a major, but not the sole determinant of Oncotype Dx score.

Ki 67 protein is found in proliferating cells. It is present in the nuclei of cells in G1, S, G2, and M phases of the cell cycle. Ki 67 protein levels are low during G1- and early S-phase, and gradually increase to reach a maximum during mitosis. Therefore, Ki 67 protein expression can be a useful marker of cell proliferation (Urruticoechea *et al*, 2005).

Multiple studies have shown an association of Ki 67 expression with prognosis and response to systemic treatments in both the neoadjuvant and adjuvant settings. High-Ki 67 expression has been associated with an increased risk of breast cancer recurrence and cancer death (Lee *et al*, 1997; Colleoni *et al*, 2004; Kronqvist *et al*, 2004; Penault-Llorca *et al*, 2009). Tumours with high Ki 67 before neoadjuvant chemotherapy have a higher rate of pathologic response, and lower Ki 67 after neoadjuvant chemotherapy is associated with favourable disease-free survival (DFS; Nishimura *et al*, 2010; Yerushalmi *et al*, 2010). At least two studies have suggested that patients with high-Ki 67 tumours may benefit more from adjuvant chemotherapy and endocrine therapy (Viale *et al*, 2008a, 2008b; Penault-Llorca *et al*, 2009).

The Oncotype Dx gene test (Genomic Health, Redwood City, CA, USA) is a commercially available reverse transcriptase PCR assay (RT–PCR) of 21 genes, which uses a specific algorithm to calculate the recurrence score (RS) for oestrogen receptor (ER)-positive breast cancers. On the basis of RS, patients are considered low risk (RS<18), intermediate risk (18⩽RS<31), or high risk (RS⩾31). Although different cancer-related genes including ER and HER-2 expression are included in calculating the RS, five proliferation genes (*Ki 67, STK 15, Survivin, CCNB 1*, and *MYBL 2*) are heavily weighted and especially important in this calculation (Paik *et al*, 2004). This test is currently used for predicting the risk of recurrence in lymph node-negative ER-positive breast cancers, and is considered by the National Comprehensive Cancer Network guidelines as an option to help decision-making in this group of patients. Unfortunately, the high cost of this multigene assay limits its’ use in daily practice in many countries.

The aim of this study was to compare the correlation of immunohistologically measured Ki 67 protein expression with the Oncotype Dx RS in patients with lymph node-negative ER/progesterone receptor (PR)-positive, HER-2-negative breast cancers.

## Materials And Methods

With approval from the Institutional Research Ethics Committee, we analysed 53 cases of T1–2 N0 M0 (ER/PR-positive, HER-2-negative) breast cancer treated in the Jewish General Hospital, Montreal, Canada. These cases were chosen randomly from a pool of patients with early-stage breast cancer, who had Oncotype Dx analysis of their tumour. Pathology reports were reviewed and histological type, tumour size, Nottingham grade, perineural invasion status, lymphovascular invasion status, invasive tumour necrosis, ER levels, and PR levels were recorded. ER and PR levels were recorded as per the Allred Score (Harvey *et al*, 1999). All tumours were HER-2 negative, using immunohistochemistry (IHC) or fluorescent *in situ* hybridisation. Sentinel lymph node sampling data were available in all patients and no lymph node involvement was reported. The Oncotype Dx RS results were provided by Genomic Health test reports. Patients were stratified to three risk groups as per Oncotype RS: low risk (RS<18), intermediate risk (18⩽RS<31), and high risk (RS⩾31) (Paik *et al*, 2004).

Ki 67 expression was examined by IHC of formalin-fixed deparaffinised tissue, using prediluted rabbit monoclonal antibody against human Ki 67 (Clone 30-9, Ventana, Tucson, AZ, USA) at a concentration of 2 *μ*g ml^−1^. Slides were stained on automated immunostainer Benchmark XT from Ventana, using the iView DAB Detection Kit. The same tissue blocks were used for both Oncotype Dx testing and Ki 67 immunostaining. Results were assessed without the use of an image analysis system. The fraction of positive cells (in percentage) with definite nuclear immunostaining, including mild, moderate, and strong was counted. The representative fields were chosen at low magnification and included at least two areas at the most cellular edges of tumour, and one area in the centre. The number of cells counted at high-power magnification varied depending on distribution of Ki 67 immunopositive staining. For cases with even distribution, the Ki 67 staining was determined with 400–600 tumoural cells, but in cases with uneven distribution, up to 2500 tumoural cells were counted. The stained slides were evaluated by two of the authors when the distribution of Ki-67-positive tumoural cells was uneven, or when the percentage of immunoreactivity was near the cut-off points. Patients were divided into low-risk (Ki 67<10%), intermediate-risk (10%⩽Ki 67<25%), and high-risk group (Ki 67⩾25%) on the basis of the expression of Ki 67.

Ki 67<10% was considered low, based on a cut-off point used by Kronqvist *et al* (2004) and Breast International Group Trial 1-98 (Viale *et al*, 2008a, 2008b). However, as there were other studies that used Ki 67>20% as their cut-off point, we decided to consider Ki 67⩾25% as high (Colleoni *et al*, 2004; Viale *et al*, 2008a, 2008b). By doing so, we could study the group of patients with Ki 67 values falling between these two numbers (Ki 67 intermediate group).

The SPSS version 19 (Chicago, IL, USA) was used for statistical analysis. Linear regression, univariate analysis, multivariate analysis, and partial correlation analysis were performed.

## Results

The pathologic characteristics of patients are presented in Table 1.

**Figure 1:**
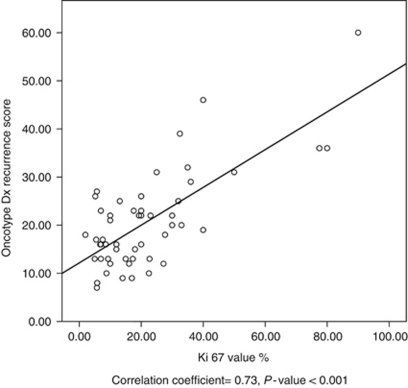
Correlation of Oncotype DX RS with Ki 67 value. Correlation coefficient=0.73, *P*-value<0.001.

The median Ki 67 value was 17.3% (range 2–90%). The median Oncotype RS was 18 (range 7–60). There was a strong linear correlation between Ki 67 expression and Oncotype RS (correlation coefficient=0.73, *P*-value<0.001; Figure 1).

There was also a significant correlation between Nottingham grade and Oncotype RS on univariate analysis (correlation coefficient=0.52, *P*-value<0.001). The correlation of Ki 67 and Oncotype RS remained robust (correlation coefficient=0.6, *P*-value<0.001), even after controlling for the effect of Nottingham grade by using partial correlation analysis and multivariate analysis. On the other hand, there was no significant correlation between Nottingham grade and Oncotype RS when the effect of Ki 67 was controlled (correlation coefficient=0.064, *P*-value=0.65). This suggests that the correlation found between Nottingham grade and Oncotype RS on univariate analysis was most likely due to the effect of Ki 67.

We also analysed the correlation between other histopathological characteristics of tumour with Oncotype RS. Previously, Flanagan *et al* (2008) reported significant correlation between nuclear grade, mitotic count, ER score, and PR score . In our study, there was a weak but significant correlation between nuclear grade and Oncotype RS (correlation coefficient=0.39, *P*-value=0.005). This correlation was weaker but still significant, when the effect of Ki 67 was controlled by using partial correlation analysis (correlation coefficient=0.32, *P*-value=0.047). There was a significant correlation between mitosis score (measured as part of Nottingham scoring) and Oncotype RS in univariate analysis (correlation coefficient=0.39, *P*-value=0.005). This correlation was not significant when the effect of Ki 67 was controlled in multivariate analysis and partial analysis (correlation coefficient=0.054, *P*-value=0.714).

In previous studies, lower expression of PR has been associated with higher Oncotype RS (Flanagan *et al*, 2008; Tang *et al*, 2010). Tang *et al* (2010) demonstrated that among ER-positive tumours, PR-poor tumours had significantly higher Oncotype RS. Our results were consistent with these findings. ER/PR expression had a significant inverse correlation with Oncotype RS, but did not affect the correlation between Ki 67 and Oncotype RS. Lower expression of PR was associated with higher Oncotype RS (correlation coefficient=−0.56, *P*-value<0.001), which was independent from the effect of ER expression. The combination of ER/PR expression and Ki 67 had a very strong correlation with Oncotype RS (correlation coefficient=0.84, (0.75–0.93) *P*-value<0.001).

There was no significant correlation between perineural invasion, lymphovascular invasion, invasive tumour necrosis, and Oncotype RS on multivariate analysis.

Most patients (93.85%) in the high-Ki 67 group (Ki 67⩾25%) had Oncotype RS of high or intermediate risk. All patients in low-Ki 67 group (Ki 67 of <10%) had RS of low or intermediate risk (Table 2).

## Discussion

Oncotype Dx testing provides valuable prognostic and predictive information in patients with early-stage breast cancers. Unfortunately, the high price limits the accessibility of this test to all patients. Hence, there has been increasing interest to find simple pathology tests, which can help predict the recurrence of disease. As *Ki 67* is one of the proliferation genes assessed routinely by IHC in different malignancies, several investigators have studied its prognostic and predictive value in different stages of breast cancer treatment. High-Ki 67 expression detected by IHC has been reported as the strongest individual prognostic factor of breast cancer death or recurrence in patients with T1 N0 M0 disease (Kronqvist *et al*, 2004). Ki 67 positivity in more than 10% of cancer cells increased the odds of breast cancer death or relapse by 11-fold (Kronqvist *et al*, 2004). In another study, the 4-year DFS was lower for patients with T1 N0 M0 breast cancer and Ki 67 value ⩾20%, compared with T1 N0 M0 breast cancer and Ki 67<20% 93.3% *vs* 99.2%, respectively (Colleoni *et al*, 2004).

Ki 67 value has also been considered as a strong predictive factor for effectiveness of neoadjuvant and adjuvant systemic therapy. In a study by Nishimura *et al* (2010), pathological response after neoadjuvant chemotherapy was significantly associated with Ki 67 values. A higher pathological complete response rate was found in patients with higher Ki 67 values, and there was no pathological response in patients with Ki 67 less than 25%. Ki 67 after neoadjuvant treatment was predictive of DFS. The lower Ki 67 (<12%) was associated with favourable DFS (Nishimura *et al*, 2010). Expression of Ki 67 in ER-positive breast cancer patients has been associated with benefit from docetaxel treatment in the adjuvant setting; 5-year DFS was higher in patients with ER-positive/Ki 67-positive tumours (84%) *vs* ER-positive/Ki 67-negative tumours (81%) when they were treated with adjuvant docetaxel (Penault-Llorca *et al*, 2009). Greater benefit of adjuvant endocrine therapy with Letrozole *vs* Tamoxifen has been observed in patients with Ki 67 value>10% (Viale *et al*, 2008a, 2008b).

Ki 67 has been also found to be highly effective in dividing ER-positive tumours into luminal A and luminal B subtypes (Hugh *et al*, 2009).

One of the limitations of the use of Ki 67 value is the lack of a standardised scoring system, which should be addressed, as multiple studies, including ours, have underlined the potential use of this test in decision-making.

Our study reveals a strong correlation between Ki 67 value and Oncotype DX RS, especially in tumours with Ki 67 value ⩾25%. Our findings strengthen two recent publications by Gwin *et al* (2009) and Allison *et al* (2011). Gwin *et al* (2009) studied the association between Ki 67 value and Oncotype Dx RS in a series of 32 patients with lymph node-negative ER/PR-positive, HER-2-negative breast cancer. Although no correlation analysis was presented in this article, overall association was found between Ki 67 value and Oncotype RS in most cases. They also noticed unexpectedly high-Ki 67 value in patients with low RS. One of the limitations of the study was the fact that none of the 32 patients had high RS. On the basis of these findings, the authors suggested the combination of Oncotype DX and Ki 67 values for identifying tumours with a high potential of recurrence (Gwin *et al*, 2009). In addition, recently, Ki 67 was significantly associated with Oncotype RS, Nottingham grade, and angiolymphatic invasion (Williams *et al*, 2011).

Previously, Flanagan *et al* (2008) had shown a significant correlation between Nottingham grade and Oncotype RS (correlation coefficient=0.59, *P*<0.01). In a recently published article by Allison *et al* (2011), the correlation of Ki 67 and routine pathological parameters with Oncotype RS was studied. In this study, Nottingham grade had the strongest overall association with Oncotype RS and was used in combination with the PR level to divide cases into different subgroups.

A unique finding in our study is the fact that the correlation between Nottingham score and Oncotype RS disappeared when the effect of Ki 67 value was controlled. Our findings suggest that Ki 67 is the major driver of the correlation found between Nottingham grade and Oncotype RS.

We also found that the likelihood of a tumour with Ki 67⩾25% having a high or intermediate Oncotype RS is >90%, and these patients may be the group that benefit the most from adjuvant chemotherapy.

In summary, our data suggests that Ki 67 is the major but not the sole determinant of Oncotype RS. It will be of great interest to study an immunopanel consisting of Ki 67 with other proliferation markers measured by Oncotype Dx, that is, STK 15, Survivin, CCNB1, and MYBL2.

## Figures and Tables

**Table 1:** Pathological characteristics of tumours

	**Number (%)**
*Size*	
⩽1 cm	6 (11.3)
>1 cm	47 (88.7)
	
*Histology*	
Ductal	48 (90.6)
Lobular	5 (9.4)
	
*Nottingham grade*	
1	15 (28.3)
2	29 (54.7)
3	9 (17)
	
*Ki 67*	
Low (<10)	16 (30.2)
Intermediate (10⩽Ki 67<25)	21 (39.6)
High (Ki 67⩾25)	16 (30.2)
	
*ER level (Allred score)*	
Negative (<3)	0 (0)
Weak (3–4)	1 (1.9)
Strong (⩾5)	52
	
*PR level (Allred score)*	
Negative (<3)	6 (11.3)
Weak (3–4)	9 (17)
Strong (⩾5)	38 (71.7)
	
*Oncotype Dx RS category*	
Low (RS⩽17)	25 (47.2)
Intermediate (18⩽RS⩽30)	20 (37.7)
High (RS⩾31)	8 (15.1)

Abbreviations: ER=oestrogen receptor; PR=progesterone receptor; RS=recurrence score.

**Table 2:** Distribution of cases by Ki 67 value and Oncotype Dx RS

	**Low-oncotype RS (%)**	**Intermediate- oncotype RS (%)**	**High-oncotype RS (%)**
Low Ki 67	12 (75)	4 (25)	0 (0)
Intermediate Ki 67	12 (57.1)	9 (42.9)	0 (0)
High Ki 67	1 (6.25)	7 (43.85)	8 (50)

Abbreviation: RS=recurrence score.
